# The effect of the participatory heat education and awareness tools (HEAT) intervention on agricultural worker physiological heat strain: results from a parallel, comparison, group randomized study

**DOI:** 10.1186/s12889-022-14144-2

**Published:** 2022-09-15

**Authors:** Erica Chavez Santos, June T. Spector, Jared Egbert, Jennifer Krenz, Paul D. Sampson, Pablo Palmández, Elizabeth Torres, Maria Blancas, Jose Carmona, Jihoon Jung, John C. Flunker

**Affiliations:** 1 Department of Health Systems and Population Health, University of Washington, Seattle, WA USA; 2 Department of Environmental and Occupational Health Sciences, University of Washington, 4225 Roosevelt Way NE, Seattle, WA 98105 USA; 3 Department of Medicine, University of Washington, 4225 Roosevelt Way NE, Seattle, WA 98105 USA; 4 Department of Preventive Medicine, Madigan Army Medical Center, Joint Base Lewis-McChord, Seattle, WA USA; 5 Department of Statistics, University of Washington, Seattle, WA USA; 6 Northwest Communities Education Center/Radio KDNA, Granger, WA USA

**Keywords:** Agricultural workers, Core body temperature, Heat-related illness, Heat strain, Heat stress, Heat education and awareness tools (HEAT), Intervention study, Physiological strain index

## Abstract

**Background:**

Farmworkers are at risk of heat-related illness (HRI). We sought to: 1) evaluate the effectiveness of farmworker Spanish/English participatory heat education and a supervisor decision-support mobile application (HEAT intervention) on physiological heat strain; and 2) describe factors associated with HRI symptoms reporting.

**Methods:**

We conducted a parallel, comparison group intervention study from May–September of 2019 in Central/Eastern Washington State, USA. We used convenience sampling to recruit adult outdoor farmworkers and allocated participating crews to intervention (*n* = 37 participants) and alternative-training comparison (*n* = 38 participants) groups. We measured heat strain monthly using heart rate and estimated core body temperature to compute the maximum work-shift physiological strain index (PSI_max_) and assessed self-reported HRI symptoms using a weekly survey. Multivariable linear mixed effects models were used to assess associations of the HEAT intervention with PSI_max_, and bivariate mixed models were used to describe factors associated with HRI symptoms reported (0, 1, 2+ symptoms), with random effects for workers.

**Results:**

We observed larger decreases in PSI_max_ in the intervention versus comparison group for higher work exertion levels (categorized as low, low/medium-low, and high effort), after adjustment for maximum work-shift ambient Heat Index (HI_max_), but this was not statistically significant (interaction − 0.91 for high versus low/medium-low effort, t = − 1.60, *p* = 0.11). We observed a higher PSI_max_ with high versus low/medium-low effort (main effect 1.96, t = 3.81, *p* < 0.001) and a lower PSI_max_ with older age (− 0.03, t = − 2.95, *p* = 0.004), after covariate adjustment. There was no clear relationship between PSI_max_ and the number of HRI symptoms reported. Reporting more symptoms was associated with older age, higher HI_max_, 10+ years agricultural work, not being an H-2A guest worker, and walking > 3 min to get to the toilet at work.

**Conclusions:**

Effort level should be addressed in heat management plans, for example through work/rest cycles, rotation, and pacing, in addition to education and other factors that influence heat stress. Both symptoms and indicators of physiological heat strain should be monitored, if possible, during periods of high heat stress to increase the sensitivity of early HRI detection and prevention. Structural barriers to HRI prevention must also be addressed.

**Trial registration:**

ClinicalTrials.gov Registration Number: NCT04234802, date first posted 21/01/2020.

**Supplementary Information:**

The online version contains supplementary material available at 10.1186/s12889-022-14144-2.

## Background

Heat exposure is associated with substantial occupational mortality and morbidity, including from heat-related illness (HRI), traumatic injuries, and acute kidney injury [1–5]. In 2015, exposure to heat caused 2830 occupational injuries and illnesses resulting in days away from work and 37 work-related deaths in the United States (US), 89% of which occurred during the summer months (June–September) [6]. Agricultural workers have high rates of HRI and heat-related deaths. From 2000 to 2010, agricultural workers had more than 35 times the risk of heat-related death compared to other industry sectors, with a yearly average fatality rate of 3.1 per 1 million workers [1]. In the agriculturally intensive State of Washington (WA), there were a total of 918 workers’ compensation HRI claims during 2006–2017, with the agriculture, forestry, fishing, and hunting sector having the second highest third quarter (July–September) rate (102.6 claims per 100,000 full-time employees [FTE]) and the highest annual HRI claims rate (13.0 per 100,000 FTE) [7]. HRIs are likely more prevalent than data indicate [7, 8], as less severe injuries and illnesses may be self-treated and not reported to supervisors, and agricultural workers may prioritize work over taking time off for treatment and recuperation [9]. The risk of HRI is unlikely to diminish in the future, as the frequency and intensity of heat events is projected to increase [10].

Field evaluations of the effectiveness of interventions to reduce farmworker HRI risk are needed to support prioritization of the most promising approaches. Though there is growing evidence that farmworker education that is participatory, culturally and linguistically appropriate, and tailored to agriculture is effective in improving heat knowledge and behavioral intentions [11, 12], few studies have investigated the effectiveness of these interventions on objective measures of heat strain. Pilot evaluations of the effectiveness of different cooling strategies and hydration on core body temperature and kidney function among agricultural workers have been performed [13, 14]. Formative work suggests that supervisor mobile applications that provide local weather conditions and recommendations for protecting workers from heat may be acceptable to agricultural supervisors [15, 16]. A mobile application that provides users with information about predicted heat stress based on environmental conditions, activity level, clothing, and acclimatization has also been developed and evaluated [17]. Interventions that include an emphasis on water, rest, and shade at work have shown promise, including in preventing adverse heat health effects among sugarcane workers in Central America [18]. California, WA, and Oregon are the only three US states that have developed emergency or permanent occupational heat rules intended to prevent outdoor HRI [19–22]. However, research in California suggests an increased risk of HRI even when farms follow California/Occupational Safety Administration heat regulations [23], suggesting that the way in which rules and practices are implemented and the effectiveness of specific provisions needs further evaluation. Risk factors for adverse heat health effects exist at multiple levels (e.g., individual, co-worker, employer, community, and policy levels), yet few studies have developed interventions using a multi-level framework tailored to agricultural settings [3].

Heat stress is defined within the American Conference of Governmental Industrial Hygienists (ACGIH) Threshold Limit Value (TLV)® as the net heat load to which a worker may be exposed from the combined contributions of metabolic heat (e.g., from physical work), environmental factors, and clothing [24]. Heat strain refers to the overall physiological response to heat stress aimed at dissipating excess heat from the body, and the TLV aims to maintain the core body temperature within 1 °C of normal (37 °C) [24]. HRIs include heat rash, heat exhaustion, heat syncope (fainting), and heat stroke, which is associated with an elevated core body temperature (> 40 °C, 104 °F) and can be fatal. Different HRIs manifest clinically with different groups of symptoms. Though occupational health guidelines and rules incorporate recognition and reporting of HRI symptoms [19–22, 24–26], HRI symptoms may be non-specific (e.g., headache, fatigue), there is little consensus on how best to categorize HRI symptoms [27] or how reporting of symptoms relates to physiological heat strain, and different factors may affect reporting of HRI symptoms. Physiological monitoring of heat strain does not rely on self-report and captures individual responses to heat load, which depend on several factors, including personal factors (e.g., age, sex, fitness level, acclimatization status, health conditions, medications, hydration level), environmental conditions, workload, and clothing [26].

Agricultural workers are integral to the US food supply, and there are opportunities to improve agricultural worker safety and health. In this study, our primary objective was to evaluate the effectiveness of a multi-level HRI prevention approach that addresses individual, community, and employer level factors through worker education and a supervisor decision support mobile application among agricultural workers in WA. We hypothesize that this multi-level Heat Education and Awareness Tools (HEAT) intervention can improve HRI awareness and prevention practices and therefore reduce physiological heat strain among agricultural workers. Our secondary objective was to describe the relationship between objectively measured physiological heat strain and self-reported symptoms and to describe factors associated with HRI symptoms reporting.

## Methods

### Study design and setting

This study, the HEAT intervention study, is a parallel, comparison, group randomized intervention study to evaluate the effectiveness of a multi-level HEAT intervention approach for agricultural workers and supervisors that includes: 1) worker education; and 2) a heat awareness mobile application (HEAT App) that informs supervisors of hot conditions during the coming week and provides recommendations to keep workers safe [28]. The study took place in 2019 in agriculturally intensive areas of Central/Eastern WA, where tree fruit, cherries, and other crops such as grapes and hops are predominant [29]. Eastern WA is characterized by warmer and drier summers than Western WA, with average summer high temperatures in the upper 80s to mid-90s°F (27–34 °C) [30]. The study took place from May–September, as the majority of hot days in WA occur between May and September. Baseline survey data and initial rounds of weekly symptoms data collection began in May. Field data collection occurred from June to August, and the final round of weekly symptom data was collected in September. Agricultural workers in Central/Eastern WA are largely Latinx/e and include seasonal workers and US H-2A guest workers. Latinx or Latine are non-binary and neutral forms of Latinos, and they are used to acknowledge marginalized and excluded members of the diverse Latinx/e community [31–33]**.** The US H-2A program is a federal program that allows employers to hire workers on temporary work permits from other countries for agricultural jobs [34]. The University of Washington Human Subjects Division (HSD) approved all study procedures, and participants provided written informed consent prior to study participation.

### Intervention development

Study details and information about HEAT intervention development have been previously reported [28]. In brief, the HEAT intervention was developed in collaboration with regional agricultural stakeholders and communities through long-standing partnerships with Pacific Northwest Agricultural Safety and Health (PNASH) Center researchers. Intervention development was grounded in the social-ecological model of prevention [28, 35, 36] and guided by two advisory groups: 1) a technical advisory group, which included agricultural industry, government, and community representatives; and 2) an expert working group, which included farmworkers and managers [28]. Research staff included individuals who live and work in agricultural communities in WA. The HEAT intervention was designed to cover factors that affect HRI risk at multiple levels, including the individual, workplace, and community levels [28].

The first intervention component, HEAT education, was developed to be culturally and linguistically appropriate and tailored to agriculture and uses a relational and engaged approach in the language of preference of the target audience (Spanish or English) [28]. HEAT education includes a Spanish/English train-the-trainer facilitator’s guide, uses poster visual displays, and covers: 1) types of HRI and treatments; 2) risk factors for HRI; 3) staying hydrated at work; 4) clothing for work in hot weather; 5) personal protective equipment and heat; and 6) keeping cool in the home and community [37]. HEAT education was designed to comply with WA’s Outdoor Heat Rule for Agriculture worker training requirements [20]. Feedback from advisory groups, results from focus groups and beta testing with promotores (community health workers) and agricultural workers, which involved providing early versions of the HEAT education and making adjustments based on feedback, and guidance from the University of Washington Center for Teaching and Learning were used to refine the HEAT education materials [28]. The entire training guide takes approximately 60–90 minutes to complete but can also be broken down into 15-minute toolbox trainings for use in the field. Our prior study of HEAT education among WA farmworkers found greater improvement in worker heat knowledge scores across a summer season in the HEAT intervention group, compared to a comparison group that was offered non-HRI alternative training (*p* = 0.04) [12].

The second intervention component, the HEAT App, was developed in partnership with Washington State University’s AgWeatherNet (AWN) Program. AWN maintains a network of over 200 professional weather stations located mostly in agriculturally productive regions of Central/Eastern WA and is a trusted source of weather information for crop decision support in the WA agricultural community [38]. The HEAT App links current and forecasted weather information with health and safety messages. HEAT App development was grounded in elements of the Technology Acceptance Model [28, 39], and the HEAT App was designed to notify agricultural supervisors about hot weather conditions and send messages through push notifications. Messages contain information about workers’ risk for adverse health effects from heat and strategies for prevention that are tailored to the agricultural industry (Fig. S1). As previously described [28], messages are sent one and 6 days before a forecasted Heat Index of 91 °F (33 °C) or higher at nearby weather stations selected by the user. Suggested actions for heat prevention are available for conditions between a Heat Index of 80–90 °F (27–32 °C), but push notifications are not sent out below 91 °F (99 °C) to avoid information overload.

### Recruitment & eligibility

We used convenience sampling to recruit participants from agricultural companies from Central/Eastern WA in the late Spring 2019, as previously described [28]. There were a total of four tree fruit and vineyard companies that agreed to participate. The research team provided information sessions about the study and recruited participants from participating employers’ crews. There were approximately two to six crews per participating company from which crews were recruited. Crews were already formed by the workplace, and researchers did not have the ability to assemble crews. As described in the Intervention allocation section below, crews within large and small companies were allocated to intervention and comparison groups separately, as large and small companies differ in their capacity for dedicated health and safety personnel and programs. Two of the four companies, hereafter referred to as ‘Large-1′ and ‘Large-2,’ were considered large companies, with more than 50 full-time employees during the growing season and dedicated health and safety personnel. We enrolled two crews from each large company for a total of four crews (Fig. S2). The other two companies had less than 50 full-time employees and were considered small companies. Since the two small companies were owned by brothers and had similar safety and health practices, the two small companies were considered one company, hereafter referred to as ‘Small,’ for the purposes of the analysis. We enrolled two crews from ‘Small’ for a total of two crews (Fig. S2). This recruitment strategy yielded ‘Large-1′, ‘Large-2′, and ‘Small’ enrolled companies and six enrolled crews (two per company) (Fig. S2), with eight to 17 participants per crew. Eligible participants included seasonal workers and US H-2A guest workers, workers aged 18 years or older, workers who planned to work in agriculture during the summer season, and workers who understood Spanish and/or English.

### Intervention allocation

Research staff were trained to use simple randomization (coin flip) to randomly allocate crews of participating workers within each company to intervention and comparison groups. Workers and supervisors were not provided with information about which group they were allocated to, but researchers were aware of group allocation. One crew from each company was assigned to the intervention group (three crews total) and the other crew from each company to the comparison group (three crews total) (Fig. S2). Due to logistical constraints related to the timing of agricultural work, crews from ‘Small’ were not randomized; the first to enroll received the intervention, and the second was assigned to the comparison group. All participants were offered the intervention after data collection was complete.

### Study procedures & flow

After obtaining informed consent, workers were asked to complete a baseline survey in Spanish or English. Work characteristics, including company, crew, and H-2A status, were noted by field staff on field observation sheets. Workers in the HEAT intervention group then received HEAT education from the same research staff member. Workers in the comparison group were offered education on another topic of interest to them (e.g., sexual harassment, pesticides). The HEAT App was provided in Spanish or English to intervention group supervisors who directly supervised each crew over the course of the season. Research staff assisted intervention group supervisors in downloading the application to their mobile device, selecting weather stations closest to their worksites, and viewing current heat indices and maximum daily heat indices forecasted over the following week. Approximately monthly, research staff conducted field monitoring, including field observations, surveys, and physiological monitoring at the farm (see Data collection and processing below). Participants were also asked to complete a weekly symptoms survey via a mobile phone application or phone call.

**Fig. 1:**
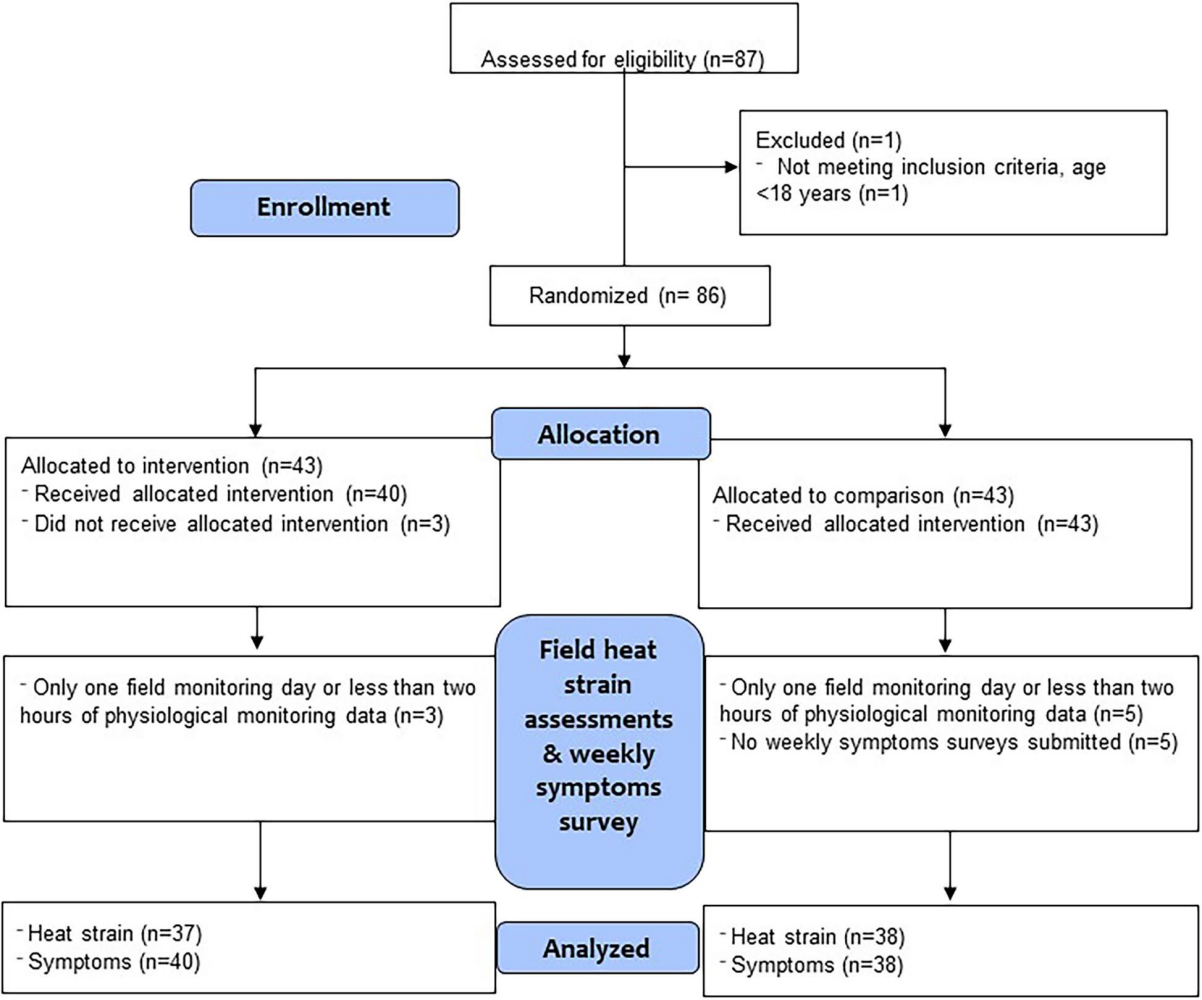
Study flow

Details of the study flow are shown in Fig. 1. Overall, 87 participants were evaluated for eligibility. One participant was excluded because they were ineligible (age less than 18 years), and therefore 86 participants from six crews were enrolled. Three participants allocated to the intervention group did not receive the intervention and were excluded. Three and five participants did not have more than one field monitoring day or at least 2 hours of physiological heat strain data in the intervention and comparison groups, respectively, and were excluded from the primary analysis of the relationship between the HEAT intervention and heat strain. A total of 75 participants were available for the primary analysis of heat strain. Five participants did not have available weekly symptoms survey data and were additionally excluded from secondary analyses of the relationship between heat strain and symptoms and from descriptive analyses of factors associated with HRI symptoms reporting.

### Data collection & processing

#### Baseline survey

Participants completed the baseline survey on paper or a computer tablet in Spanish or English, depending on the participant’s preference (Fig. S3). Spanish/English bicultural/bilingual study staff members were available to read the questions and response choices to the participants, as needed. The baseline survey consisted of 42 questions covering years of experience working in agriculture, distance to toilet at work, previous HRI training, medical conditions, cooling practices, and demographic information (e.g., age, sex, country of origin, years in the US). The baseline survey and the weekly symptoms survey, discussed in the next section, were based on our previous survey, which has been evaluated for validity and reliability in a similar population, as previously described [40].

#### Weekly symptoms survey

A weekly Spanish/English check-in survey was administered to participants at the end of every week, on Thursday-Sunday, excluding holidays, throughout the study period (Fig. S4). The survey asked about the previous 7 days of work. Participants had the option to complete the survey using a smartphone application (LifeData, LLC; Marion, IN) that sent a notification to complete the survey on Thursday afternoon with subsequent reminders on Friday. Participants who did not complete the survey using the phone application, as well as those that did not feel comfortable filling out the survey using the application, were called every week on Friday by a bilingual/bicultural research team member and asked the survey questions. Participants who did not answer or did not have time to complete the survey by Friday were called on Saturday or Sunday. The weekly check-in survey was designed to take approximately 5 minutes and included questions about HRI symptoms, including: 1) skin rash or skin bumps, 2) painful muscle cramps or spasms, 3) dizziness or light-headedness, 4) fainting, 5) headache, 6) nausea or vomiting, 7) heavy sweating, 8) extreme weakness and fatigue, and 9) confusion.

#### Physiological strain index (PSI)

Our primary outcome was physiological heat strain (PSI). We measured tympanic temperatures using tympanic thermometers (Braun; Kronberg, Germany) at the beginning of the work-shift on field monitoring days. Baseline core temperature (T_0_) was estimated by adding 0.27 °C to the tympanic temperature to account for differences between tympanic temperature and core body temperature [41]. Research staff assessed baseline heart rates (HR_0_) by asking participants to rest for approximately 10 minutes and taking participants’ radial pulses for 15 seconds, then multiplying by four, in the morning before work shifts. Workers’ heart rates were logged every 20 seconds throughout the work-shift using Polar® chest band monitors (Polar, Inc.; Lake Success, NY). Heart rate measurements below 40 beats per minute were removed, as these values were considered outside of the physiologically expected range. Only one participant had 39 minutes of nonzero heart rate measurements below 40 beats per minute on 1 day, and these values were excluded. No participants had heart rates above 200 beats per minute. One-minute average heart rates (HR_x_) were then computed. We employed a US Army Research Institute of Environmental Medicine (USARIEM) method [42], which uses an extended Kalman filter algorithm, to produce estimates of core body temperature every minute (T_x_) from one-minute heart rate measurements (HR_x_) and baseline core body temperature (T_0_). This algorithm has been validated in military settings and evaluated among WA agricultural workers [43]. We calculated PSI using the equation PSI = 5*[(T_x_ -T_0_)/(39.5-T_0_)] + 5*[(HR_x_-HR_0_)/(180-HR_0_)] [44]. A higher PSI indicates higher heat strain.

#### Body mass index

Participant height and weight were measured on field observation days. Due to work demands, participants did not always have time to take off their work boots prior to measurements. If this was the case, shoes were accounted for by subtracting five pounds from the weight and one inch from the height measurements. Height and weight measurements were used to calculate body mass index (BMI) [kg/m^2^] [45]. BMI was included in analyses because it may be associated with HRI risk [46].

#### Heat index

For the primary heat strain analysis, research staff recorded work start and end times on field observation days. We obtained data on air temperature and relative humidity during the work shift from nearby AWN stations, which log data in 15-minute intervals [38]. We selected the two closest weather stations on observation days from each known work area, resulting in the inclusion of stations within 8000 m of each known work area. We used Rothfusz’s modification of Steadman’s work to calculate the Heat Index from temperature and relative humidity [47, 48]. Values from included weather stations for each crew on each observation day were averaged. For each participant, we trimmed data to work start and end times. Data were then summarized per participant to generate maximum daily Heat Index (HI_max_) values on observation days.

#### Effort level

Field research staff recorded participant task and crop observations on field data sheets. Based on field observations and review of crop and task combinations by study team members with training in occupational safety and health, we used the main observed task to generate the following effort categories: high = tree fruit harvest (there was no grape harvest during field observation days); medium-high = digging holes, fixing posts, installing wire (tree fruit), tying branches (tree fruit), uncovering trees, tree fruit pruning, tree fruit thinning; medium-low = weeding, grape thinning, irrigation, tying branches (grapes), installing wire (grapes); low = using tractor, driving car, welding. If more than one task was recorded as the main task, the task with the maximum effort level was used to determine the effort category. For the analysis, low and medium-low categories were combined together (low/medium-low).

### Statistical analyses

We used descriptive univariate and bivariate statistics, box plots, and scatter plots to characterize participant baseline characteristics and time-varying characteristics of effort level, HI_max_, and PSI.

#### Association of HEAT intervention with PSI

The repeated or longitudinal assessments of participants requires an analysis method that accounts for correlation among these repeated measurements. We therefore assessed the association of maximum work shift PSI (PSI_max_) with group status (intervention versus comparison, with group assigned using intention-to-treat) using linear mixed effects models with random effects for workers. Although our power analysis [28] did not take into account covariates, as prior information on the effects of all covariates was not available, we report two models to demonstrate how the apparent intervention effect on PSI_max_ is modified by two factors described extensively in the literature to be associated with heat strain (effort level and Heat Index) [49, 50], and then how all these effects are modified by adjustment for demographic factors. We present Model 1, which accounts for HI_max_ centered around the mean (degrees Fahrenheit), effort level (low/medium-low [reference category], medium-high, and high), and the interactions of effort level with HI_max_ and group. We hypothesized that the effect of the intervention may be greater among those with higher compared to lower effort levels. We also present Model 2, which accounts for the following potential confounders: 1) individual: age (years), sex (female [reference category], male), and BMI (kg/m^2^); 2) work: effort level, HI_max_, and company (small [reference category], large-1, and large-2); and 3) terms for the interaction of effort level with HI_max_ and group. We do not report an interaction of group status with HI_max_ as the modest sample size does not support meaningful (significant) estimation of possible variation of an intervention effect with heat exposure in addition to its variation with effort level. The nominal significance (*p*-values) for the 2-degrees of freedom terms involving the 3-level coding of effort were computed using the lmerTest package in R [51].

#### Relationship of PSI with HRI symptoms reported & factors associated with HRI symptoms reporting

We coded the symptoms variable as an ordinal variable: no symptoms reported (0), one symptom reported (1), and two or more symptoms reported (2+). We used box plots to visualize the relationship between PSI_max_ and HRI symptoms reported. To describe the relationship of factors other than PSI_max_ associated with HRI symptoms reporting (ordinal), we used bivariate descriptive statistics and mixed models with random effects for workers using the clmm2 function in the ordinal package in R.

All analyses were conducted using RStudio Server Version 1.4.1717 [52].

## Results

### Baseline survey

Baseline characteristics of the study population are shown in Table 1**.** About two-thirds of participants (77%) were between 25 and 64 years of age. Over half of participants reported primary school or less education (51%) and living in the US for more than 10 years (55%). Ninety-six percent of participants reported being born in Mexico. Forty-three percent of participants reported working in agriculture in the US for 10 or more years, and 37 % of participants reported being H-2A workers. About one-fifth (21%) of participants reported being told by a healthcare provider of having high blood pressure, but only 7 and 3% reported being told by a healthcare provider of having diabetes mellitus and heart disease, respectively. The mean (standard deviation) BMI was 30.2 (5.0) kg/m^2^.

**Table 1:** Baseline characteristics by intervention versus comparison group (n [%] or mean [sd])

Characteristic	All (***N*** = 75)	Comparison (***n*** = 38)	Intervention (***n*** = 37)
**Age (years)**			
18–24	10 (13%)	5 (13%)	5 (14%)
25–44	30 (40%)	14 (37%)	16 (43%)
45–64	28 (37%)	15 (39%)	13 (35%)
> 64	7 (9%)	4 (11%)	3 (8%)
**Sex**			
Male	48 (64%)	21 (55%)	27 (73%)
Female	27 (36%)	17 (45%)	10 (27%)
**Education level**			
Primary school or less	38 (51%)	20 (53%)	18 (49%)
Some or all of middle school	9 (12%)	5 (13%)	4 (11%)
Some or all of high school	22 (29%)	9 (24%)	13 (35%)
More than high school	5 (7%)	4 (11%)	1 (3%)
Don’t know/refused/missing	1 (1%)	0 (0%)	1 (3%)
**Years living in the US**			
< 1	25 (33%)	12 (32%)	13 (35%)
3–10	7 (9%)	2 (5%)	5 (14%)
> 10	41 (55%)	23 (61%)	18 (49%)
Don’t know/refused/missing	2 (3%)	1 (3%)	1 (3%)
**Country of origin**			
United States	2 (3%)	0 (0%)	2 (5%)
Mexico	72 (96%)	38 (100%)	34 (92%)
Don’t know/refused/missing/other	1 (1%)	0 (0%)	1 (3%)
**Years working in agriculture in the US**			
< 1	29 (39%)	16 (42%)	13 (35%)
1–5	8 (11%)	4 (11%)	4 (11%)
6–9	6 (8%)	1 (3%)	5 (14%)
10 or more	32 (43%)	17 (45%)	15 (41%)
**H-2A status**			
H-2A worker	28 (37%)	14 (37%)	14 (38%)
Not H-2A	47 (63%)	24 (63%)	23 (62%)
**Company**			
Small	15 (20%)	8 (21%)	7 (19%)
Large - 1	28 (37%)	14 (37%)	14 (38%)
Large - 2	32 (43%)	16 (42%)	16 (43%)
**Received heat-related illness training in the past year**			
Yes	54 (72%)	24 (63%)	30 (81%)
No	20 (27%)	14 (37%)	6 (16%)
Don’t know/refused/missing	1 (1%)	0 (0%)	1 (3%)
**Informed by healthcare provider about selected personal health conditions**^**a**^			
Diabetes	5 (7%)	3 (8%)	2 (5%)
High blood pressure	16 (21%)	9 (24%)	7 (18%)
Heart disease	2 (3%)	2 (5%)	0 (0%)
**Body mass index** (kg/m^2^)	30.2 (5.0)	30.1 (5.2)	29.7 (4.8)

^a^Categories not mutually exclusive

In general, the distribution of participant baseline characteristics was well balanced between comparison and intervention groups. However, 73% of participants in the intervention group were male compared to 55% in the comparison group, and 81% of participants in the intervention group reported receiving HRI training in the past year compared to 63% in the comparison group.

Forty-three percent of participants worked in the Large-2 company, 37% worked in the Large-1 company, and 20% of participants worked in the Small company (Table 1). Participants from the Small company participated in field observations in July and August, participants from the Large-1 company participated in field observations mostly in June but also in July and August, and participants from the Large-2 company participated in field observations nearly evenly across June, July, and August (Table S1).

### Heat exposure and outcomes

**Fig. 2:**
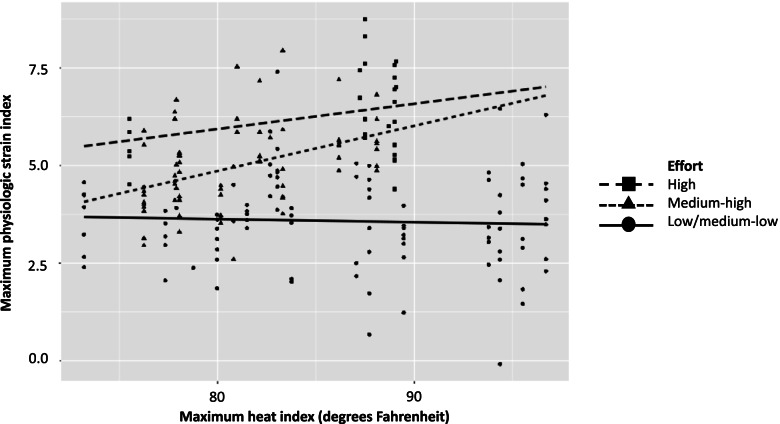
Scatter plot of maximum Heat Index and PSI by effort level. Lines are unadjusted regression lines using linear models

**Fig. 3:**
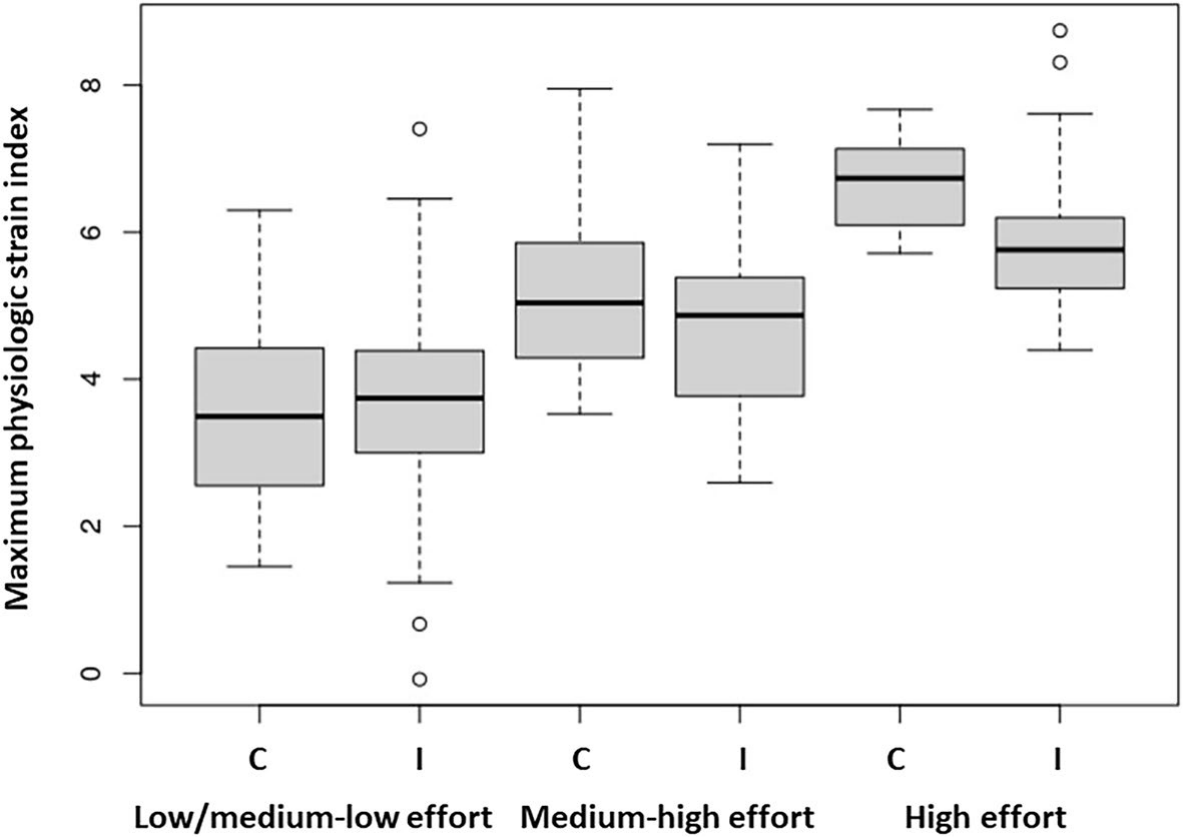
Box plot of maximum PSI by effort and group status. C = comparison; I = intervention groups

The mean (standard deviation) PSI_max_ was 4.3 (1.5) in the intervention group and 4.6 (1.5) in the comparison group. The mean HI_max_ and mean PSI_max_ by month and group are shown in Table 2. In general, the monthly mean PSI_max_ and mean HI_max_ were higher in the comparison group compared to the intervention group. The relationship between HI_max_ and PSI_max_ by effort level is shown in Fig. 2. Higher PSIs_max_ are seen with higher effort, and an increase in PSI_max_ with increasing HI_max_ is seen for high and medium-high effort but not for low/medium-low effort. A greater difference in median PSI_max_ is seen between the intervention and comparison groups with increasing effort, with a notably higher median PSI_max_ in the comparison compared to the intervention group in the highest effort category (Fig. 3).

**Table 2:** Mean max PSI and mean max HI by month and group (mean, sd)

Month	Group	Mean (sd) max PSI	Mean (sd) max HI (°F)	Mean (sd) max HI (°C)
June	Intervention	4.6 (1.4)	83.8 (5.4)	28.8 (3.0)
	Comparison	4.8 (1.5)	85.4 (5.4)	29.7 (3.0)
July	Intervention	3.7 (1.4)	83.4 (7.0)	28.5 (3.9)
	Comparison	4.0 (1.2)	83.7 (7.1)	28.7 (3.9)
August	Intervention	4.4 (1.8)	82.4 (6.0)	28.0 (3.3)
	Comparison	5.0 (1.6)	87.4 (7.6)	30.8 (4.2)

### Association of HEAT intervention with PSI

Results of linear mixed effects models of PSI_max_ are shown in Table 3. In Model 1, effort modified the effect of HI_max_ on PSI_max_ (*p* = 0.01), with an increase in the HI_max_ slope of 0.11 PSI_max_ units per degree Fahrenheit (one degree Celsius is 1.8 times larger than one degree Fahrenheit) for medium-high effort compared to low/medium-low effort (t = 2.71, *P* = 0.01). The intervention by effort interaction terms show the high effort group having an average comparison-intervention difference of 0.91 PSI_max_ greater than that in the low/medium-low group, but this estimated effect is not significant (t = − 1.60, *p* = 0.11). In Model 2, in which we additionally accounted for company, age, gender, and BMI, there was a 0.03 unit decrease in PSI_max_ per year of age (t = − 2.95, *p* = 0.004) and an increase in PSI_max_ of 0.04 per 1.0 kg/m^2^ increase in BMI (t = 2.05, *p* = 0.04). The magnitudes of the two interaction effects are reduced and rendered less significant after accounting for differences among the companies and demographics. Models 1 and 2 showed a larger decrease in PSI_max_ in the intervention compared to the comparison group for higher effort levels, but this was not formally statistically significant.

**Table 3:** Main effects and interaction effects of linear mixed effects models of maximum PSI^a^

	Model 1	Model 2
Group *(ref. comparison)*	0.26 (0.30), t = 0.87	0.08 (0.27), t = 0.31
Max Heat Index (HI_max_)^b^	0.01 (0.02), t = 0.77	0.02 (0.02), t = 1.25
Effort *(ref. low/medium-low)*		
medium-high	2.01 (0.33), **t = 6.03**	0.87 (0.43), t = 2.03
high	2.99 (0.45), **t = 6.63**	1.96 (0.51), **t = 3.81**
Company *(ref. small)*		
large-1	–	−0.01 (0.42), t = − 0.02
large-2	–	− 0.73 (0.51), t = − 1.44
Age	*–*	− 0.03 (0.01), **t = −2.95**
Sex *(ref. female)*	*–*	− 0.14 (0.28), t = − 0.49
BMI	*–*	0.04 (0.02), **t = 2.05**
**Interactions**		
Group*effort	*P* = 0.21	*P* = 0.39
medium-high	−0.61 (0.44), t = −1.40	− 0.37 (0.40), t = − 0.92
high	−0.91 (0.57), t = − 1.60	−0.67 (0.53), t = − 1.27
HI_max_^b^ *effort	P = 0.01	*P* = 0.10
medium-high	0.11 (0.04), **t = 2.71**	0.08 (0.04), t = 1.91
high	0.08 (0.05), t = 1.79	0.05 (0.04), t = 1.19

^a^t-thresholds for nominally significant effects at p-values of 0.05, 0.01, and 0.001 are +/− 1.64, 2.58, and 3.29, respectively, and indicated in **bold**

^b^Heat Index was centered at the mean value

### Relationship of PSI with HRI symptoms reported & factors associated with HRI symptoms reporting

**Fig. 4:**
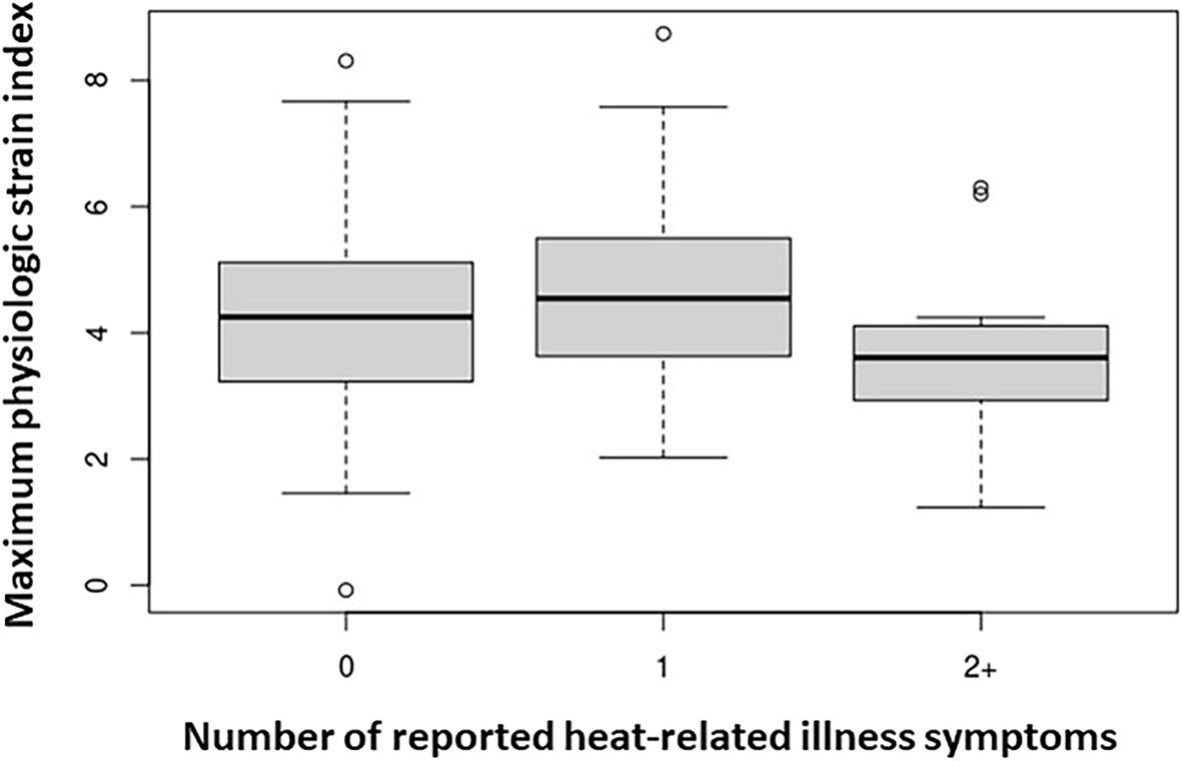
Box plot of maximum PSI by number of reported HRI symptoms^a^. ^a^N = 65 unique participants with PSI and HRI symptoms data within a week of field PSI measurement, 121 observations

A box plot of PSI_max_ with number of reported HRI symptoms is shown in Fig. 4. There is no clear relationship between PSI_max_ and the number of reported HRI symptoms. Results of reported HRI symptoms by participant characteristics are shown in Table 4**.** Participants were more likely to report more symptoms if they were older, working in agriculture in the US for 10 or more years, not H-2A workers, reported a longer walk to get to the toilet at work, experienced a higher HI_max_ at work, and reported having cooling opportunities in the community or air conditioning at home.

**Table 4:** Participant characteristics by number of reported heat-related illness symptoms, and results from bivariate mixed models^a^

		Number of symptoms reported			Coefficients^**b**^ (standard errors) and Z values^**c**^ from bivariate mixed models
Characteristic	Total (***N*** = 76^**a**^, 554 observations)	0 (382 obs)	1 (126 obs)	2+ (46 obs)	
**Age (years)**					
Mean (SD)	41.7 (15.3)	39.6 (15.1)	45.6 (14.9)	48.8 (13.9)	0.04 (0.02), **2.25**
**Sex**					
Female	183 (100%)	112 (61.2%)	50 (27.3%)	21 (11.5%)	ref
Male	371 (100%)	270 (72.8%)	76 (20.5%)	25 (6.7%)	−0.89 (0.55), −1.62
**Years working in agriculture in the US**					
< 1	241 (100%)	196 (81.3%)	38 (15.8%)	7 (2.9%)	ref
1–5	53 (100%)	42 (79.2%)	7 (13.2%)	4 (7.5%)	0.25 (0.86), 0.29
6–9	41 (100%)	28 (68.3%)	13 (31.7%)	0 (0.0%)	0.79 (0.93), 0.85
10 or more	219 (100%)	116 (53.0%)	68 (31.1%)	35 (16.0%)	1.83 (0.53), **3.45**
**H-2A Status**					
H-2A worker	238 (100%)	189 (79.4%)	42 (17.6%)	7 (3.0%)	ref
Not H-2A	316 (100%)	193 (61.1%)	84 (26.6%)	39 (12.3%)	1.34 (0.54), **2.46**
**Company**					
Small	90 (100%)	65 (72.2%)	20 (22.2%)	5 (5.6%)	ref
Large - 1	238 (100%)	189 (79.4%)	42 (17.6%)	7 (2.9%)	−0.60 (0.56), −1.07
Large - 2	226 (100%)	128 (56.6%)	64 (28.3%)	34 (15.0%)	1.07 (0.67), 1.60
**Distance to toilet**					
3 min or less	383 (100%)	278 (72.6%)	83 (21.7%)	22 (5.7%)	ref
> 3 min	147 (100%)	81 (55.1%)	42 (28.6%)	24 (16.3%)	1.33 (0.55), **2.41**
Missing	24 (100%)	23 (95.8%)	1 (4.2%)	0 (0.0%)	–
**Previous HRI training**					
No	140 (100%)	106 (75.7%)	27 (19.3%)	7 (5.2%)	ref
Yes	397 (100%)	264 (66.5%)	94 (23.7%)	39 (9.8%)	0.29 (0.61), 0.46
Missing	17 (100%)	12 (70.6%)	5 (29.4%)	0 (0.0%)	–
**Effort level**					
Low/medium-low	349 (100%)	227 (65.0%)	83 (23.8%)	39 (11.2%)	ref
Medium-high	91 (100%)	69 (75.8%)	18 (19.8%)	4 (4.4%)	−1.03 (0.52), **−1.98**
High	100 (100%)	74 (74.0%)	23 (23.0%)	3 (3.0%)	−0.17 (0.37), −0.47
Missing	14 (100%)	12 (85.7%)	2 (14.3%)	0 (0.0%)	–
**Mean of weekly max Heat Index**^**d**^					
Mean (SD)	83.4 (5.1)	83.3 (5.1)	83.1 (5.4)	85.3 (4.1)	0.06 (0.02), **2.38**
**Cooling opportunities of outside work/air conditioning at home**					
No	198 (100%)	158 (79.8%)	29 (14.6%)	11 (5.6%)	Ref
Yes	356 (100%)	224 (62.9%)	97 (27.2%)	35 (9.8%)	1.08 (0.55), **1.97**
**Previous heat-related illness**					
No	495 (100%)	342 (69.1%)	113 (22.8%)	40 (8.1%)	Ref
Yes	7 (100%)	3 (42.9%)	4 (57.1%)	0 (0.0%)	1.27 (2.06), 0.61
Missing	52 (100%)	37 (71.2%)	9 (17.3%)	6 (11.5%)	–
**Body mass index** (kg/m^2^)^e^					
Mean (SD)	29.8 (5.0)	29.7 (5.2)	30.3 (4.9)	28.7 (4.1)	0.01 (0.05), 0.18

^a^Two participants excluded because of missing weather data

^b^Coefficients are interpreted as: compared to reference category (for categorical variables), or for a one unit increase (continuous variables), there is change in the log odds of 1+ or 2+ symptoms by the value of the coefficient

^c^Z-thresholds for nominally significant effects at p-values of 0.05, 0.01, and 0.001 are +/− 1.64, 2.58, and 3.29, respectively, and indicated in **bold**

^d^We selected the closest AWN weather stations within 8000 m of known work locations. Values from included weather stations were averaged. For each participant, we trimmed data to work start and end times and to the reported days working in the past week

^e^Four observations missing

## Discussion

We conducted a parallel, comparison group intervention study of the HEAT intervention, consisting of culturally- and agriculture-tailored participatory farmworker heat education and a supervisor decision-support mobile application, in Washington State, US. We found larger decreases in physiological heat strain across a summer season in the intervention compared to comparison group for higher levels of work exertion, but this was not statistically significant. Prior studies suggest that participatory education that is culturally tailored is associated with improved farmworker heat knowledge and behavioral intentions [11, 12] and that mobile heat safety and decision support applications are well-received by agricultural supervisors [16]. However, knowledge and behavioral intentions alone may not lead to change. According to the Health Belief Model, behavior change is achieved through targeting perceived barriers, benefits, susceptibility, and threats [53]. While tailored participatory education and supervisor decision support could influence these factors, findings from our study support the principle that reductions in heat strain and the risk of HRI require additional elements of heat stress management at the workplace level and systemic change to address barriers to reporting symptoms, pay structure, and access to healthcare.

The strongest predictor of physiological strain in this study was work exertion (effort level). Results from our fully adjusted model indicate that workers performing tasks requiring high effort had PSI_max_ levels almost two points higher, on average, than those performing low/medium-low effort tasks. In our study, high effort corresponds roughly to moderate-high metabolic rate activities (300–415 W) while low/medium-low effort corresponds roughly to light-moderate tasks (180–300 W), depending on the specifics of the task [24]. This is consistent with other studies among California and Florida agricultural workers that have identified work rate and physical activity level to be associated with elevated core body temperatures [49, 50]. Contributors to heat stress include metabolic heat (e.g., from physical work), environmental factors, and clothing [24]. Participants in this study generally wore long-sleeved shirts underneath hooded sweatshirts or button-down shirts and long pants. PSI is a function of heart rate and core body temperature, and the PSI_max_ for participants performing low effort work was largely in the low range (PSI 3–4), as defined by Moran et al. [44], compared to the medium PSI range (PSI 5–6) or higher for participants performing high effort work. It is possible that in WA during the study period, when the mean HI_max_ were in the 80s, metabolic heat was a key driver of heat stress and subsequent heat strain. This is consistent with 2006–2017 WA workers’ compensation HRI claims observations that indicate that the maximum daily temperatures on illness days were below the current WA heat rule temperature threshold of 89 °F (32 °C) for 45% of claims [7]. Overall, given the strong effect of exertion on PSI, further emphasis is needed to ensure adequate rest breaks, job rotation, and/or work pace reduction in the heat, along with payment for breaks and other mechanisms to reduce financial barriers to cool down and rest.

There are several potential reasons for the observed lack of a statistically significant HEAT intervention effect. First, the sample size of this study may not have been adequate to detect effects in fully adjusted models, including interactions. Second, our estimates of the intervention effect may have been an underestimate. It is possible there was sharing of information between intervention and comparison workers, which may have led to more conservative estimates of the effectiveness of the intervention, as the analysis was intention-to-treat. For example, at one of the large companies (Large-1), crews sometimes lived and worked together. Finally, though HEAT education has been shown to result in significant improvement in worker knowledge compared to comparison crew workers [12], worker education alone may not lead to actions to reduce heat strain and the risk of HRI. Unlike the existing US Occupational Safety & Health Administration (OSHA)/National Institute for Occupational Safety & Health (NIOSH) Heat Index-based mobile heat application [54, 55], our HEAT App provides messages tailored to agriculture, local environmental data from nearby agricultural weather stations, and longer forecasting for work planning, in response to early advisor and expert working group feedback. However, in contrast to NIOSH and ACGIH heat safety guidelines, the OSHA/NIOSH and our HEAT applications are simpler tools that don’t explicitly include work pace, clothing, and acclimatization status into risk calculations and may not fully represent risk, particularly at high workloads [26]. Education and decision support should be combined with other factors in heat stress management plans and policies, including behavioral thermoregulation and sufficient rest breaks, acclimatization procedures, adequate hydration, clothing, emergency response procedures, and consideration of mechanization and work pace.

We did not find a clear relationship between PSI and the number of symptoms reported. These findings are consistent with previous reports of heat stroke occurring in the absence of reported symptoms, and symptoms occurring in the absence of other signs of HRI [26]. We were unable to distinguish whether the number of symptoms reported was the number of actual symptoms experienced or was influenced by factors affecting willingness to report. Factors associated with PSI and factors associated with the number of symptoms reported were not the same. Participants were more likely to report symptoms if they were older in age and worked in agriculture in the US for 10+ years, which may reflect actual increased symptoms, awareness and perception of symptoms, or comfort with reporting. This is different to findings from our 2013 survey study of 97 WA tree fruit harvest workers, which indicated a lower odds of reported HRI symptoms with increasing age [40]. However, the present study sample included 37% H-2A workers, who, based on our observations, tended to be younger, male, and perform work requiring higher exertion levels than our 2013 study, which may have been reflected in higher PSI_max_ values. Our fully adjusted models of heat strain indicated that PSI_max_ scores decreased almost ½ point per decade of age, on average, in contrast with the increase in symptom reporting with older age. H-2A workers in our study were less likely to report HRI symptoms, consistent with prior studies [56]. H-2A workers often do not have adequate access to healthcare, and barriers to reporting may include fear of reporting affecting current and future employment and well-being [57]. Additional work is needed to ensure adequate healthcare for H-2A and other agricultural workers and to address concerns about retaliation for reporting.

Several other factors were associated with symptoms reporting. First, in contrast to a previous study of Florida agricultural workers, women in our study were not significantly more likely to report a higher number of HRI symptoms [27]. In WA, between 2006 and 2017, there was a higher percentage of males with accepted HRI State Fund workers’ compensation claims, compared to all State Fund claims (82% versus 68%, respectively, *P* < 0.001) [7]. Though this may reflect employment predominance in agriculture by males, our study raises the question of whether differential reporting by men and women may also influence these results. Second, participants were more likely to report symptoms if they reported cooling opportunities outside of work or air conditioning at home. It is difficult to determine whether these participants seek cooling opportunities because they are more likely to experience symptoms or vice versa. There was no significant relationship between prior HRI training and symptoms reporting. Participants were more likely to report symptoms when exposed to hotter work conditions and having to walk more than three minutes to get to the toilet at work. A longer distance to the toilet has been previously reported to be associated with HRI symptoms [40]. Farmworkers, particularly those paid by the amount harvested (piece rate), may be less likely to fully hydrate and urinate regularly if it requires a longer time away from work to walk to the toilet. Piece-rate pay has been previously reported to be associated with reported HRI symptoms and with acute kidney injury among agricultural workers [40, 58]. Well-maintained toilets that are mobile, for example, toilets attached to trucks that move with workers, and consideration of payment schemes that do not incentivize skipping breaks, but still allow workers to maintain their well-being, may support optimal hydration and HRI risk reduction.

### Strengths & limitations

Strengths of this study include: its controlled, comparison design; recruitment and monitoring of agricultural workers and supervisors, including H-2A workers, over a harvest season; participatory design of the research and intervention; and collection of objective physiological data on heat strain, in addition to subjective symptom reporting. In addition to the small sample size and potential for cross-over previously discussed, this study has several other important limitations. First, we used convenience sampling in the selection of companies and workers. It is possible that companies and workers who chose not to participate may have had a greater potential for improvement in heat strain with the intervention, rendering our results more conservative. Second, weekly symptoms reporting may have been subject to recall bias. Third, due to time constraints and work pressures, the small farm crews were not randomized, and the crew that arrived first was allocated to the intervention. However, we do not have reason to believe that there are systematic differences in crews by their arrival time. Fourth, some participants completed weekly symptoms surveys by mobile application and some by telephone. Those that completed the surveys by mobile application versus telephone may have been more or less likely to report symptoms, but we do not expect there would be any systematic differences by intervention versus comparison group. Fifth, not all companies’ workers participated at the same time during the study period. Though it would be ideal to have participation on the exact same dates by all companies and workers, we were able to account for time-varying variability in factors such as heat exposure, and also accounted for company, in our analyses. Fifth, we used HI rather than more complex environmental metrics that also account for solar radiation and wind speed and that correlate better with heat strain [59]. However, we were able to assess physiologic heat strain directly, using estimated core body temperature, in our analyses. Though solar radiation has been reported to affect certain heat-related body responses [60], we do not expect that there was differential variability in solar radiation in the intervention and comparison groups. Sixth, we did not use a published compendium of physical activities to categorize task [61]. However, we did use expert review of crop and task combinations by study team members with training in occupational safety and health and who observed tasks in the field. Seventh, we used an approach to estimate core body temperature rather than assessing gastrointestinal temperature. However, this approach has been evaluated among WA agricultural workers and demonstrated to correlate well with gastrointestinal temperature [43]. Finally, our study may not be generalizable to agricultural populations in other states, as it was conducted in a US Pacific Northwest State with an occupational heat rule [20].

## Conclusion

In this study of the HEAT intervention among Washington State, US farmworkers, we found larger decreases in physiological heat strain across a summer season in the intervention compared to comparison group for higher levels of work exertion, but this was not statistically significant. The strongest predictor of physiological strain in this study was work exertion (effort level). In addition to education and administrative controls, other factors that affect heat stress, including effort level, clothing, hydration, acclimatization, and emergency response plans, must be directly addressed in workplace heat management plans to prevent excessive physiological heat strain and its effects. Effort level can be addressed through work/rest cycles, job rotation, and adjustment of work pace.

In our study, work and worker characteristics associated with heat strain and HRI symptoms reporting did not fully overlap. Physiological strain and reported HRI symptoms should not be assumed to be overlapping outcomes for the purposes of evaluating heat prevention interventions for farmworkers. Additional work is needed to understand factors that affect farmworker HRI symptoms reporting and to establish a consensus on specific HRI symptoms for monitoring purposes. During periods of high heat stress, symptoms and indicators of physiological heat strain should both be monitored, if possible, to increase the sensitivity of early HRI detection and prevention for farmworkers. Related structural issues relevant to barriers to reporting symptoms, pay structure, restrooms, and access to healthcare are also critical to consider in reducing risk of HRI for farmworkers.

These multi-level issues should be addressed using a multi-level approach at workplace, community, and individual levels, including through policy development and through the implementation of policies and best practices tools in a manner tailored for agricultural workers. The need for further action is becoming increasingly urgent as the frequency and severity of heat events are projected to increase in the future [10, 62].

## Supplementary Information

**Additional file 1.**

## Data Availability

The datasets generated and analyzed during the current study are not publicly available, as they are human data, and open availability could compromise participant privacy. However, de-identified data and statistical code will be made available from the corresponding author on reasonable request.

## Abbreviations

AWN: AgWeatherNet
ACGIH: American Conference of Governmental Industrial Hygienists
BMI: Body Mass Index
FTE: Full-Time Employees
HEAT: Heat Education and Awareness Tools
HI: Heat Index
HR: Heart Rate
HRI: Heat-Related Illness
HSD: Human Subjects Division
NIOSH: National Institute for Occupational Safety & Health
OSHA: Occupational Safety & Health Administration
PNASH: Pacific Northwest Agricultural Safety and Health
PSI: Physiological Heat Strain
SD: Standard Deviation
T: Temperature
TLV: Threshold Limit Value
US: United States
WA: Washington State
