# Correction: The effect of the participatory Heat Education and Awareness Tools (HEAT) intervention on agricultural worker physiological heat strain: results from a parallel, comparison, group allocated study

**DOI:** 10.1186/s12889-026-26808-4

**Published:** 2026-03-27

**Authors:** Erica Chavez, June T. Spector, Jared Egbert, Jennifer Krenz, Paul D. Sampson, Pablo Palmández, Elizabeth Torres, Maria Blancas, Jose Carmona, Jihoon Jung, John C. Flunker

**Affiliations:** 1 Department of Health Systems and Population Health, University of Washington, Seattle, WA USA; 2 Department of Environmental and Occupational Health Sciences, University of Washington, 4225 Roosevelt Way NE, Seattle, WA 98105 USA; 3 Department of Medicine, University of Washington, 4225 Roosevelt Way NE, Seattle, WA 98105 USA; 4 Department of Preventive Medicine, Madigan Army Medical Center, Joint Base Lewis- McChord, Seattle, WA USA; 5 Department of Statistics, University of Washington, Seattle, WA USA; 6 Northwest Communities Education Center/Radio KDNA, Granger, WA USA

**Correction: BMC Public Health 22**,** 1746 (2022)**

**https://doi.org/10.1186/s12889-022-14144-2**

Following publication of the original article [1], the authors reported an error in the article title.

The updated article title is provided below and the changes have been highlighted in **bold**.

The effect of the participatory Heat Education and Awareness Tools (HEAT) intervention on agricultural worker physiological heat strain: results from a parallel, comparison, **group allocated** study.

The original article [1] has been updated.
