# Supplementary material for: The effect of the participatory heat education and awareness tools (HEAT) intervention on agricultural worker physiological heat strain: results from a parallel, comparison, group randomized study
Source: BMC Public Health. 2022 Sep 15;22:1746. doi: 10.1186/s12889-022-14144-2 (PMC9476265; doi:10.1186/s12889-022-14144-2)
Supplement: Supplementary file 1 — Additional file 1. [file 12889_2022_14144_MOESM1_ESM.docx]

**Figure S1.** HEAT mobile application messages

| **Forecast high heat index** | **Category** | **Suggested actions** |
| --- | --- | --- |
| <80 | Minimal risk | No suggested actions for the heat |
| 80-90 | Low risk | **HYDRATION**  Clean, labeled water jugs with cool fresh water near workers  Encourage workers to regularly sip water  **COMMUNICATE WITH WORKERS**  Review emergency response procedures  Watch out for heat illness symptoms, self and co-workers  Employee inform supervisor if not feeling well  **WORK ORGANIZATION**  Assemble shade structures if no existing shade  Employees use “buddy system”, don’t work alone |
| 91-103 | Moderate risk | **HYDRATION**  Clean, labeled water jugs with cool fresh water near workers  Drink **at least** 16 oz water per hour, double as it gets hotter  Port-a-potties 3-minute walk from work, separate from fresh water  **COMMUNICATE WITH WORKERS**  Review emergency response procedures  Watch for heat illness symptoms, self and co-workers  Employee inform supervisor if not feeling well  Wear light-colored, breathable clothing, no layers, wide-brimmed hat  Consider wearing wet bandana on forehead or neck  **WORK ORGANIZATION**  Assemble shade structures if no existing shade  Employees use “buddy system”, don’t work alone  Lighter tasks, in shade, and/or frequent breaks during hotter time of day  Work in cooler time of day  Check in with workers **at least** every hour when temperatures go up  Minimize work on ladders, heights & uneven surfaces to prevent falls |
| 104-115 | High risk | ****WORK IS NOT RECOMMENDED****  **HYDRATION**  Clean, labeled water jugs with cool fresh water near workers  Water on ATV, close to workers  Drink **at least** 32 oz water per hour as it gets hot  Port-a-potties 3-minute walk from work, separate from fresh water  **COMMUNICATE WITH WORKERS**  Review emergency response procedures  Watch for heat illness symptoms, self and co-workers  Employee inform supervisor if not feeling well  Wear light-colored, breathable clothing, no layers, wide-brimmed hat  Consider wearing wet bandana on forehead or neck  **WORK ORGANIZATION**  Assemble shade structures if no existing shade  Employees use “buddy system”, don’t work alone  Lighter tasks, in shade, and/or frequent breaks during hotter time of day  Start the work day early and end by early afternoon  Work in cooler time of day  Check in with workers **at least** every 30 minutes when temperatures go up  Minimize work on ladders, heights & uneven surfaces to prevent falls |

**Figure S2.** Schematic of companies and crews for the analysis


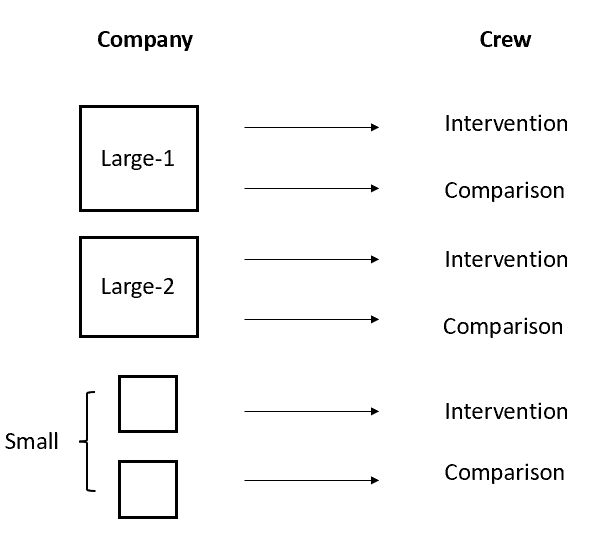


**Figure S3.** Baseline questionnaire

Heat Baseline Survey 2019

English Version

| 1) How many years have you worked in agriculture in the U.S. (including this year)?   - Less than 1 year - 1 to 2 years - 3 to 5 years - 6 to 9 years - 10 or more years - I don't know |
| --- |
| 2) When did you start working outdoors this year on orchards, vineyards, farms, or in fields?   - Before May - During the first half of May - During the last half of May - During the first half of June - I don't know |
| 3) Do you feel like you are allowed to take extra breaks if you need to rest or drink water?   - Yes - No - I don't know |

| 4) What do you usually drink at work?   - Water - Sports drinks like Gatorade or Cytomax - Energy drinks like Red Bull, Monster, or 5-hour Energy - Fruit juice - Iced coffee or iced tea - Hot coffee or hot tea - Soda - Other drinks not listed here - I don't know |
| --- |
| 5) Do you drink the amount of water you want to at work?   - Yes - No - I don't know   Why didn't you drink the amount of water you wanted to at work?   - Toilet was not nearby - Toilet was dirty - I didn't want to take a break to get a drink - I am not allowed to take a break to get a drink - I was trying to lose weight - I didn't bring any water with me - I ran out of water - Other reason - I don't know |
| 6) How long does it usually take you to walk to where there is drinking water?   - Less than one minute - Between one to three minutes - Between three to five minutes - Between five to ten minutes - More than ten minutes - There is no drinking water - I don't know |

| 7) How long does it usually take you to walk to the toilet?   - Less than one minute - Between one to three minutes - Between three to five minutes - Between five to ten minutes - More than ten minutes - There is no toilet - I don't know |
| --- |
| 8) Do you drink water provided for you at work?   - Yes - No - I don't know   Why don't you drink water provided for you at work?   - Water provided at work was too far away - Water provided at work ran out - Water provided at work was too warm - Water provided at work was too cold - Water provided at work was not clean - Other reason - I don't know |
| 9) Do you bring your own water to drink at work?   - Yes - No - I don't know   Why don't you bring your own water to drink at work?   - Not allowed - Don't want to carry water bottle - Drink water provided by work - Other reason - I don't know |

| 10) In the past week, did you remove layers or unbutton or unzip clothing when you felt hot?   - Yes - No - I don't know |
| --- |
| 11) In the past 12 months, did you receive any training about working outdoors in the heat or health effects of working in the heat?   - Yes - No - I don't know |
| 12) Do you have other paid jobs (in addition to your regular farm job) that require physical work?   - Yes - No - I don't know |
| 13) Has a doctor or other health provider ever told you that you have any of the following conditions?   - Diabetes - High blood pressure - Heart disease - Lung disease, including asthma - Overweight or obese - Heat-related illness - Kidney disease - Liver disease - High cholesterol - Conditions affecting balance, including stroke and problems with the inner ear - Sleep problems, including obstructive sleep apnea - Cancer - No, I do not have any of these medical conditions - I don't know |

| 14) Do you participate in any physical activities such as running, soccer, gardening, or walking for exercise?   - Yes - No - I don't know |
| --- |
| 15) Have you smoked at least 100 cigarettes in your entire life? (5 packs = 100 cigarettes)   - Yes - No - I don't know |
| 16) Do you now smoke cigarettes every day, some days, or not at all?   - Every day - Some days - Not at all - I don't know |
| 17) Do you now smoke cigars or pipes every day, some days, or not at all?   - Every day - Some days - Not at all - I don't know |
| 18) Do you now chew tobacco every day, some days, or not at all?   - Every day - Some days - Not at all - I don't know |

| 19) In the past week, on how many days did you have at least one drink of any alcoholic beverage such as a beer, glass of wine, or a drink with liquor?   - 1 day - 2 days - 3 days - 4 days - 5 days - 6 days - 7 days - I did not drink any alcohol this past week - I don't know |
| --- |
| 20) In the past week, on the days when you had beer, wine, or liquor, about how many did you drink on average?   - 1 or 2 - 3 or 4 - 5 or 6 - More than 6 - I don't know |
| 21) How concerned are you about your health being affected by working in hot conditions?   - Not at all concerned - A little bit concerned - Very concerned - I do not have an opinion |
| 22) Would you say that in general your health is:   - Excellent - Very good - Good - Fair - Poor - I don't know |

| 23) Which of the following best describes your housing?   - Single family home - Apartment or duplex - Barracks or dormitory - Mobile home or trailer - Vehicle - Other - I don't know |
| --- |
| 24) Who provides your housing?   - Own home - Rented but not through work - Rented through government or charity program - Rented through employer - Provided for free by employer - Live with family or friends - Other - I don't know |
| 25) How many adults (18 and over) live in your housing?  adults |
| 26) How many children (under 18) live in your housing?  children |
| 27) How many people sleep in the same room as you?  people |
| 28) In the place where you currently live, do you have the following?   - Windows that can open - Air conditioning that works - Electric fan - None of these - I don't know |

| 29) How do you keep cool at home on hot days?   - Take cool showers or baths - Use a fan - Use air conditioning - Avoid cooking inside - Close curtains or blinds when it is hot - Open windows during cooler parts of the day - Other - I don't know |
| --- |
| 30) When you are not working, do you sometimes go somewhere other than your home to cool down during the day?   - Yes - No - I don't know |
| 31) Where do you go to cool off?   - Park - Library - Mall - Community center - Swimming pool - River or lake - Other - None - I don't know |

| 32) What year were you born?   \|  \| Before 1948 \| - D \| 1955 \| - D \| 1963 \| - D \| 1971 \| - D \| 1979 \| - D \| 1987 \| - D \| 1995 \| \| --- \| --- \| --- \| --- \| --- \| --- \| --- \| --- \| --- \| --- \| --- \| --- \| --- \| --- \| \| - D \| 1948 \| - D \| 1956 \| - D \| 1964 \| - D \| 1972 \| - D \| 1980 \| - D \| 1988 \| - D \| 1996 \| \| - D \| 1949 \| - D \| 1957 \| - D \| 1965 \| - D \| 1973 \| - D \| 1981 \| - D \| 1989 \| - D \| 1997 \| \| - D \| 1950 \| - D \| 1958 \| - D \| 1966 \| - D \| 1974 \| - D \| 1982 \| - D \| 1990 \| - D \| 1998 \| \| - D \| 1951 \| - D \| 1959 \| - D \| 1967 \| - D \| 1975 \| - D \| 1983 \| - D \| 1991 \| - D \| 1999 \| \| - D \| 1952 \| - D \| 1960 \| - D \| 1968 \| - D \| 1976 \| - D \| 1984 \| - D \| 1992 \| - D \| 2000 \| \| - D \| 1953 \| - D \| 1961 \| - D \| 1969 \| - D \| 1977 \| - D \| 1985 \| - D \| 1993 \| - D \| 2001 \| \| - D \| 1954 \| - D \| 1962 \| - D \| 1970 \| - D \| 1978 \| - D \| 1986 \| - D \| 1994 \| - D \| I  don't know \| |
| --- | --- | --- | --- | --- | --- | --- | --- | --- | --- | --- | --- | --- | --- | --- | --- | --- | --- | --- | --- | --- | --- | --- | --- | --- | --- | --- | --- | --- | --- | --- | --- | --- | --- | --- | --- | --- | --- | --- | --- | --- | --- | --- | --- | --- | --- | --- | --- | --- | --- | --- | --- | --- | --- | --- | --- | --- | --- | --- | --- | --- | --- | --- | --- | --- | --- | --- | --- | --- | --- | --- | --- | --- | --- | --- | --- | --- | --- | --- | --- | --- | --- | --- | --- | --- | --- | --- | --- | --- | --- | --- | --- | --- | --- | --- | --- | --- | --- | --- | --- | --- | --- | --- | --- | --- | --- | --- | --- | --- | --- | --- | --- | --- |
| 33) Are you male or female?   - Male - Female - Other - I don't know |
| 34) How well can you read in Spanish?   - Very well - Fairly well - Not very well - Not at all - I don't know |
| 35) How well can you read in English?   - Very well - Fairly well - Not very well - Not at all - I don't know |

| 36) What level of education did you complete?   - Part of primary school - Completed primary school - Part of middle school - Completed middle school - Part of high school - Completed high school - Part of college or university - Completed college or university - I did not go to school - I don't know |
| --- |
| 37) Which of the following categories best describes the total income for last year for the household where you live? (Include income from all sources such as wages, public assistance, investments, etc.)   - Less than $14,999 - $15,000-29,999 - $30,000-60,000 - More than $60,000 - I don't know |
| 38) What is your race?   - American Indian/Alaskan Native - Asian - Native Hawaiian or Other Pacific Islander - Black or African American - White - More than one race - I don't know |
| 39) Do you consider yourself Hispanic or Latino or Latina?   - Yes - No - I don't know |

| 40) How many years have you been living in the United States?   - Less than 1 year - 1-2 years - 3-4 years - 5-7 years - 8-10 years - More than 10 years - I don't know |
| --- |
| 41) Do you live in the United States all year?   - Yes - No - I don't know |
| 42) Where were you born?   - United States - Mexico - Central America - South America - Other - I don't know |

**Figure S4.** Weekly questionnaire

Weekly Questionnaire 2019

English Version

Did you have any of the following in the past 7 days?

- Skin rash or skin bumps
- Painful muscle cramps or spasms
- Dizziness or light-headedness
- Fainting
- Headache
- Nausea or vomiting
- Heavy sweating
- Extreme weakness and fatigue
- Confusion
- Falling from height, like a ladder
- Injury
- Burning eyes
- Runny nose
- Coughing
- Difficulty breathing
- None

Did you take any of these medications in the past 7 days?

- Pain, fever, inflammation
- High blood pressure
- Heart disease
- Mental health, like depression
- Constipation
- Irritable bowel or bladder
- Nose congestion, cough, allergies
- Pills for losing weight
- Parkinson’s disease
- Seizures
- Thyroid
- Nausea
- None

How much smoke was in the air in the past 7 days? None A little A lot

If ‘A little’ or ‘A lot’: Did your employer provide you with a mask or other protection? Yes No

If ‘A little’ or ‘A lot’: Did you bring your own mask or other protection? Yes No

Were there pesticides being applied close to where you were working in the past 7 days?

Yes No Not sure

How many days did you work in the past 7 days?

About what time did you usually start work?

About what time did you usually end work?

What crop(s) did you work with in the past 7 days?

- Grapes
- Apples
- Cherries
- Pears
- Peaches, nectarines, plums
- Hops
- Other

What were your main job tasks in the past 7 days?

*(List crops next to the job task if there is more than one crop)*

- Pick/harvest
- Weed
- Irrigate
- Plant
- Prune
- Thin blossoms or fruit
- Handle pesticides
- Sort or pack indoors
- Sort or pack outdoors
- Operate farm equipment
- Work in shop
- Supervise crew
- Other

How did you get paid?

*(List crops next to the payment type if there is more than one crop)*

- Hourly
- Piece rate (by bin, row, etc.)
- Hourly and piece rate
- By the work shift
- Other

How well did you sleep at night during the past 7 nights?

- - Very good
  - Fairly good
  - Fairly bad
  - Very bad

About how many hours did you sleep each night before work during the past 7 nights?

Have any of the following changed in the past 7 days?

- - Where you live
  - Your job
  - Your health
  - Other
  - None

**Table S1.** Number of participants by company and field observation month

| **Field observation month** |  | **June** | **July** | **August** | **Total** | |
| --- | --- | --- | --- | --- | --- | --- |
| **Small**  Intervention  Comparison |  | 0  0 | 6  4 | 5  6 |  | 11  10 |
| **Large - 1**  Intervention  Comparison |  | 26  24 | 8  12 | 10  11 |  | 44  47 |
| **Large - 2**  Intervention  Comparison |  | 15  12 | 15  13 | 13  13 |  | 43  38 |
| **Total** |  | **77** | **58** | **58** |  | **193** |
